# Macrophage activation by lipopolysaccharide, interferon-γ and interleukin-4: effect of fatty acid metabolism

**DOI:** 10.1155/S0962935195000056

**Published:** 1995-01

**Authors:** H. Darmani, J. L. Harwood, J. Parton, S. K. Jackson

**Affiliations:** 1 Department of Medical Microbiology University of Wales College of Medicine Cardiff CF4 4XN UK; 2 Department of Biochemistry University of Wales College of Cardiff Cardiff CF1 1ST UK

## Abstract

The aim of this study was to investigate the effects of interferon-γ and -β (IFN-γ, -β), interleukin-4 and -10 (IL-4, -10) and Hpopolysaccharide (LPS) on the metabolism and composition of phospholipid fatty acids in macrophages. Murine J774.2 macrophages were incubated with radiolabelled fatty acids and the appropriate stimulus and the incorporation and composition of the phospholipid classes was determined. IFN-γ and IL-4 specifically stimulated enhanced incorporation of [^14^C]-linoleic acid into the phosphatidytethanolamine fraction. IL-4 (in contrast to IFN-γ and LPS) reduced incorporation of [^14^C]- arachidonic acid into phosphatidylinositol. Incubation of J774.2 cells with linoleic acid significantly increased TNFα and nitric oxide production; arachidonic acid enhanced TNFα production but reduced nitric oxide production. It is concluded that IFN-γ, IL-4 and IL-10 may differentially regulate macrophage activation via effects on the metabolism of polyunsaturated fatty acids.


Mediators of Inflammation 4, 25–30 (1995)

### Macrophage activation by lipopolysaccharide, interferon-γ and interleukin-4: effect of fatty acid metabolism

H. Darmani,[1] J. L. Harwood,[2] J. Parton and S. K. Jackson[1,CA]

[1]Department of Medical Microbiology, University of Wales College of Medicine, Cardiff CF4 4XN and [2]Department of Biochemistry, University of Wales College of Cardiff, Cardiff CF1 1ST, UK

[CA] Corresponding Author

**The aim of this study was to investigate the effects of interferon-γ and -β (IFN-γ, -β), interleukin-4 and -10 (IL-4, -10) and lipopolysaccharide (LPS) on the metabolism and composition of phospholipid fatty acids in macrophages. Murine J774.2 macrophages were incubated with radiolabelled fatty acids and the appropriate stimulus and the incorporation and composition of the phospholipid classes was determined. IFN-γ and IL-4 specifically stimulated enhanced incorporation of [$^{14}$C]-linoleic acid into the phosphatidylethanolamine fraction. IL-4 (in contrast to IFN-γ and LPS) reduced incorporation of [$^{14}$C]-arachidonic acid into phosphatidylinositol. Incubation of J774.2 cells with linoleic acid significantly increased TNFα and nitric oxide production; arachidonic acid enhanced TNFα production but reduced nitric oxide production. It is concluded that IFN-γ, IL-4 and IL-10 may differentially regulate macrophage activation via effects on the metabolism of polyunsaturated fatty acids.**

**Key words**: Cytokines, Endotoxin, Macrophage, Membrane phospholipids, Nitric oxide, Polyunsaturated fatty acid, Tumour necrosis factor

### Introduction

Bacterial endotoxin (lipopolysaccharide, LPS) is an important inducer of the sepsis syndrome, a rapidly fatal illness which remains a major cause of morbidity and mortality in the modern medical centre. Some patients become hypersusceptible to the lethal effects of endotoxin, although the mechanism for this sensitivity remains poorly understood. Animal models of endotoxic shock have revealed that concomitant bacterial or mycobacterial infections greatly increase susceptibility to the lethal effects of endotoxin[1] and that antibody to tumour necrosis factor-α (TNFα) or interferon-γ (IFN-γ) are beneficial.[2,3] It is becoming increasingly clear that IFN-γ is an important mediator of hypersensitivity to endotoxin. IFN-γ has been shown to induce expression of LPS binding sites on the cell surface of lung macrophages which lack binding sites for LPS.[4] Furthermore, IFN-γ has been identified as the mediator of *Propionibacterium acnes*-induced LPS hypersensitivity in mice.[5]

Macrophages are primary target cells for endotoxin and by production of endogenous mediators such as superoxide anion, nitric oxide, cytokines (interleukin-1, -6, -8 (IL-1, -6, -8) and TNF) and lipid mediators (for example, prostaglandins and leukotrienes), contribute to the pathophysiology of endotoxic shock.

Infection of mice with BCG results in increases in the ratio of polyunsaturated:saturated fatty acids *in vivo* in peritoneal macrophages and it has recently been shown that IFN-γ is able to increase macrophage phospholipid polyunsaturated *in vitro*.[6,7] It is believed that this could be a possible mechanism of endotoxin sensitivity and we have demonstrated that IFN-γ and exogenous polyunsaturated fatty acids increase binding of LPS to mouse macrophages.[8] Metabolic studies are consistent with this and have demonstrated that IFN-γ may act as sensitizer to the effects of endotoxin by increasing the linoleic acid metabolism of macrophages and it has been proposed that IFN-γ could prime these cells for a heightened response to LPS.[9]

T-helper Type 1 and 2 cells are classified according to the pattern of lymphokines they produce.[10] Interferon-γ and IL-2 are produced by Th1 cells, and IL-4, IL-5 and IL-10 by Th2 cells.[11] Since these two subsets are known to be functionally distinct and frequently act antagonistically[12–15] we wish to examine the effects of IL-4 and IL-10 on the phospholipid composition of macrophage cells which are believed to be of paramount importance in the sepsis syndrome. Thus, these interleukins would form a comparison to the IFN-γ effects observed previously. In addition, IFN-β which has been shown not to play a significant role in the interaction of macrophages with endotoxin[8] was included in the experiments to confirm the specificity of any effects on fatty acid metabolism. The data reported here provide further evidence for the role of linoleate in macrophage phospholipids in mediating sensitivity to endotoxin.

### Materials and Methods

*Reagents:* All reagents were purchased from Sigma Chemical Company Ltd, Poole, UK, unless stated

© 1995 Rapid Communications of Oxford Ltd otherwise. $^{14}$C-labelled arachidonic, linoleic and stearic acids (50–60 mCi/mmol) were supplied by Amersham International plc, Aylesbury, UK. IFN-γ, IL-4 and IL-10 were purchased from Genzyme, Kent, UK.

*Cell culture:*

*Growth medium.* Cells were cultivated in Dulbecco's modification of Eagle's medium (DMEM) which had been supplemented with 10% foetal calf serum (FCS) and an antibiotic solution containing 100 units/ml penicillin and 100 μg/ml streptomycin.

*Cell line.* The murine (BALB/C) tumour monocyte–macrophage J774.2 (European Collection of Animal Cell Cultures, Salisbury, Wiltshire, UK), was the cell line used in the course of this investigation. These cells were maintained in DMEM supplemented as described above.

*Pretreatment with IFN-γ, IFN-β, LPS and interleukin-4.* Exposure of J774.2 cells (1 x $10^6$) to murine recombinant IFN-γ and IFN-β (50 U/ml), 10 μg/ml *Escherichia coli 0111:B4* LPS, and murine recombinant IL-4 and IL-10 (50 U/ml) was carried out for 18 h at 37°C, in a humidified incubator containing 5% $CO_2$. At the end of the incubation period the cells were dislodged by gentle agitation, washed three times with DMEM and collected by centrifugation at 1 000 × ***g*** for 5 min and finally resuspended in DMEM

*Preparation of ammonium salts of the radiolabelled fatty acids:* To aid solubility of the radiolabelled fatty acids, they were converted into their ammonium salts as follows. The fatty acids were supplied in either ethanol or toluene and as a first stage the solvent was evaporated under a stream of nitrogen. The ammonium salt of the fatty acid was then prepared by incubating the fatty acids in 0.2 ml of 2 M ammonia solution at 60–70°C under nitrogen for 30 min. The solution was evaporated to dryness under a stream of nitrogen and the resulting ammonium salts were resuspended in a known volume of growth medium.

*Incorporation of $^{14}$C-fatty acids:* Cells were incubated in the presence of 0.2 μCi of the radiolabelled fatty acids (ammonium salt) with or without IL-4 and IL-10 for 18 h at 37°C in a humidified $CO_2$ incubator. After the appropriate incubation time, the cells were centrifuged at 1 000 × ***g*** for 3 min, washed in 10 ml phosphate buffered saline and collected by centrifugation at 1 000 × ***g*** for 3 min and finally resuspended in 1 ml deionized water and sonicated in an ultrasonic water bath at maximum power. Once complete lysis of the cells had been achieved (verified by light microscopy) the cells were processed for phospholipid extraction.

*Extraction of phospholipids:* The method of Garbus *et al.*[16] was used since it enabled quantitation of all phospholipid classes. To 1 ml of the lysed cell suspension 3.75 ml chloroform/methanol (1:2 v/v) was added, mixed thoroughly and left at room temperature for 30 min. Then chloroform (1.25 ml) and 2 M KCl in 0.5 M phosphate buffer, pH 7.4 (1.25 ml) were added and the solution mixed thoroughly again. The chloroform phase containing the extracted phospholipids was dried in a stream of nitrogen and then subjected to thin layer chromatography.

*Thin layer chromatography:* The dried phospholipids were dissolved in 30 μl chloroform, spotted onto silica gel G plates (BDH) and chromatographed in a solvent system consisting of chloroform:methanol:acetic acid:water (50:30:8:1, by volume). Phospholipid standards were chromatographed on separate lanes. The resolved radiolabelled phospholipids were located by exposure to iodine vapour, and the appropriate areas scraped off into scintillation vials for radioactivity determination. Phospholipids to be processed for gas liquid chromatography (GLC) were located under UV light after staining with 8-anilino-1-naphthalenesulphonic acid, scraped off into sealable tubes.

*Analysis of lipid fatty acids by GLC:* Phospholipid and neutral lipid fractions were scraped from the silica gel G plates and methylated with 1 ml 2.5% $H_2SO_4$ in methanol in sealed tubes at 70°C for 2 h. The reaction was stopped by addition of 2.5 ml of 5% NaCl and the methyl esters extracted three times with petroleum ether (60–80°C fraction). Heneiconsanoic acid (21:0) was used as an internal standard and the samples were analysed using a Perkin Elmer 8600 gas chromatograph equipped with a flame ionization detector. A fused silica WCOT 50 m × 0.25 mm i.d. column coated with CP-Sil 88 was used. The column temperature was ramped from 205°C to 255°C and the injector and detector temperatures were 275°C and 305°C respectively. Carrier gas (nitrogen) was used at 20 psi. Peak areas, retention times and response factors were automatically computed, the yields being calculated using the 21:0 internal standard.

*Determination of TNFα release:* The Factor-Test-X™ Mouse TNFα ELISA kit was used to determine the concentration of TNFα released by J774.2 cells in the presence and absence of interferon-γ or -β. Briefly, 100 μl of the culture supernates were centrifuged at 1 000 × ***g*** for 2 min in order to separate any debris from the supernates and applied to a 96-well microtitre plate precoated with monoclonal anti-m-TNFα in order to capture any mTNFα present in the samples. The plate was sealed and incubated at 37°C for 2 h. The plate was then washed to remove unbound material, and a peroxidase-conjugated polyclonal anti-mTNFα (HRP-conjugate, which binds to captured mTNFα, was added and the plate was sealed and incubated for 1 h at 37°C. The plate was washed again to remove unbound material and a substrate solution was added which initiates a peroxidase catalysed colour change. The plate was incubated at room temperature for 10 min, after which time a stop solution was added which stops the colour change, by acidification. The absorbance was measured at 540 nm and this was proportional to the concentration of TNFα present in standards or samples. A standard curve was obtained by plotting the concentrations of TNFα standards *vs.* their resulting absorbances and the mTNFα concentrations in experimental samples were then determined using the standard curve.

*Nitrite assay:* One hundred μl of the culture supernates were mixed with an equal volume of Griess reagent which consisted of 1% sulfanil amide, 0.1% *N*-(1-naphthyl)-ethylenediamine dihydrochloride and 2.5% phosphoric acid and left in the dark for 20 min at room temperature. A Titertek multiscan microtitre plate reader was used for the determination of the absorbance at 540 nm, which is proportional to the concentration of nitrite in the medium.

### Results

The effects of interferons or LPS on the uptake of radiolabelled exogenous fatty acids by macrophages have recently been reported, and in this investigation we examined the effects of IL-4 and IL-10, whose biological actions are reported to contrast with some of the activating properties of IFN-γ on J774.2 cells. Uptake of $^{14}$C-linoleate and $^{14}$C-arachidonate into the major lipid classes (phosphatidylcholine (PC), PE, phosphatidylinositol (PI), and neutral lipid (NL) fractions) from macrophages were examined after pretreatment.

Figure 1a shows that IL-4 induces a statistically significant increase in the incorporation of $^{14}$C-linoleic acid into the phosphatidylethanolamine (PE) ($p < 0.01$ compared with control cells) fraction of the macrophages. The phosphatidylcholine (PC) and phosphatidylinositol (PI) fractions showed reduced incorporation ($p < 0.05$ *vs.* control cells) of this fatty acid. Figure 1b shows that IL-4 induces a small but statistically significant increase in the incorporation of $^{14}$C-arachidonic acid into the PC and a reduced incorporation into the PI fraction of the J774.2 cell membrane phospholipids.

In contrast to IL-4, IL-10 did not affect the incorporation of linoleic acid into the PE fraction. However, there was a statistically significant reduction in the incorporation of this fatty acid into the PC fraction. Furthermore, IL-10 induced an increase in the uptake of $^{14}$C-arachidonic acid into the PE fraction and a decrease in the PI (data not shown).

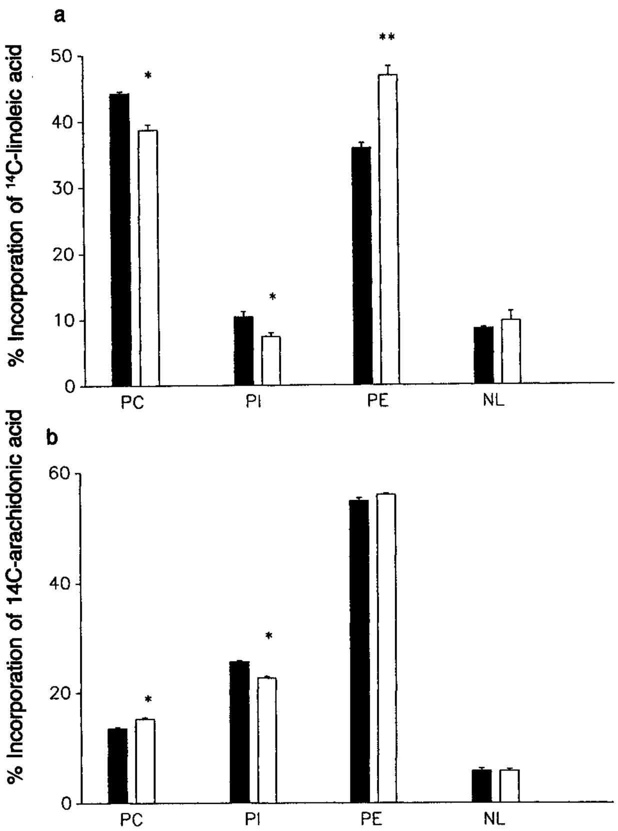

FIG. 1. Effect of IL-4 on the uptake of (a) $^{14}$C-linoleic acid and (b) $^{14}$C-arachidonic acid into the PC, PI, PE and NL fractions of J774.2 cell membrane phospholipids. Cells were incubated with 0.2 μCi of the radiolabelled fatty acids for 18 h at 37°C with and without IL-4. Error bars represent ± standard error of the means. (* $p < 0.05$; ** $p < 0.01$ *vs.* controls). ■, control; □, IL-4.

We were particularly interested in the cytokines that induce increases in the linoleate proportions of the PE fractions of J774.2 cell membrane phospholipids.[7] Therefore, the effects of IFN-γ, IFN-β, LPS and IL-4 on the endogenous linoleate, arachidonate and stearic acid content were examined.

Figure 2 shows the effects of IFN-β, LPS, IL-4 and IFN-γ on the composition of the polyunsaturated fatty acids, arachidonic acid (2a), linoleic acid (2b) and the saturated fatty acid, stearic acid (2c) of the PE fraction of the membrane phospholipids of J774.2 cells. It can be seen that IFN-γ and LPS induce increases in the content of arachidonic acid (Fig. 2a) in comparison with control cells ($p < 0.05$ *vs.* control cells for LPS treatment). The increases were 15% and 27.7%, respectively, for IFN-γ or LPS treatment. In contrast, IFN-β-pretreated cells showed a 37% decrease in the arachidonic acid content and IL-4 showed a 25% decrease ($p < 0.05$).

Figure 2b shows the percentage (by weight) of linoleic acid in the PE fraction of the membrane phospholipids of control cells and of pretreated cells. LPS, IFN-γ and IL-4 stimulated significant increases in the linoleic acid content of these cells in comparison with control cells. The greatest increases were induced by LPS and IL-4 which showed increases of

173% ($p < 0.01$ *vs.* control cells) and 212% ($p < 0.01$ *vs.* control cells) respectively. IFN-γ induced an increase of 101% ($p < 0.01$ *vs.* control cells). In contrast, IFN-β did not increase the content of linoleic acid.

As it is believed that the polyunsaturated components of the plasma membrane play an important role in predisposing to endotoxin sensitivity,[17] stearic acid composition was used as a control. IFN-γ, IL-4, LPS and IFN-β-pretreatment of the cells all result in a reduction in the percentage composition of stearic acid, in comparison with control cells (Fig. 2c). The greatest reduction in the content of this saturated fatty acid is seen in IFN-γ, LPS and IL-4-pretreated cells, which showed decreases of 68%, 48% and 38% respectively ($p < 0.001$ *vs.* control cells for all three). IFN-β caused a 24% reduction in the composition of stearic acid in the PE fraction of these cells ($p < 0.05$). Thus, these effects are in marked contrast to the differential effects of mediators on linoleate levels in J774.2 cells.

Figure 3 shows the results of the ELISA assay for TNFα release in control cells and in cells preincubated with arachidonic and linoleic acids for 18 h and subsequently incubated with LPS for 4 h. Control cells produced very little TNFα but when they were incubated with LPS for 4 h there was a great increase in the release of this cytokine ($p < 0.00001$ *vs.* control cells). Cells which had been preincubated with linoleic acid and then with LPS for 4 h showed increased TNFα release ($p < 0.05$ *vs.* control cells which were subsequently incubated with LPS). Although arachidonic acid pretreatment resulted in a slight increase in TNFα release, the increase was not statistically significant. Cells preincubated with these fatty acids without subse-

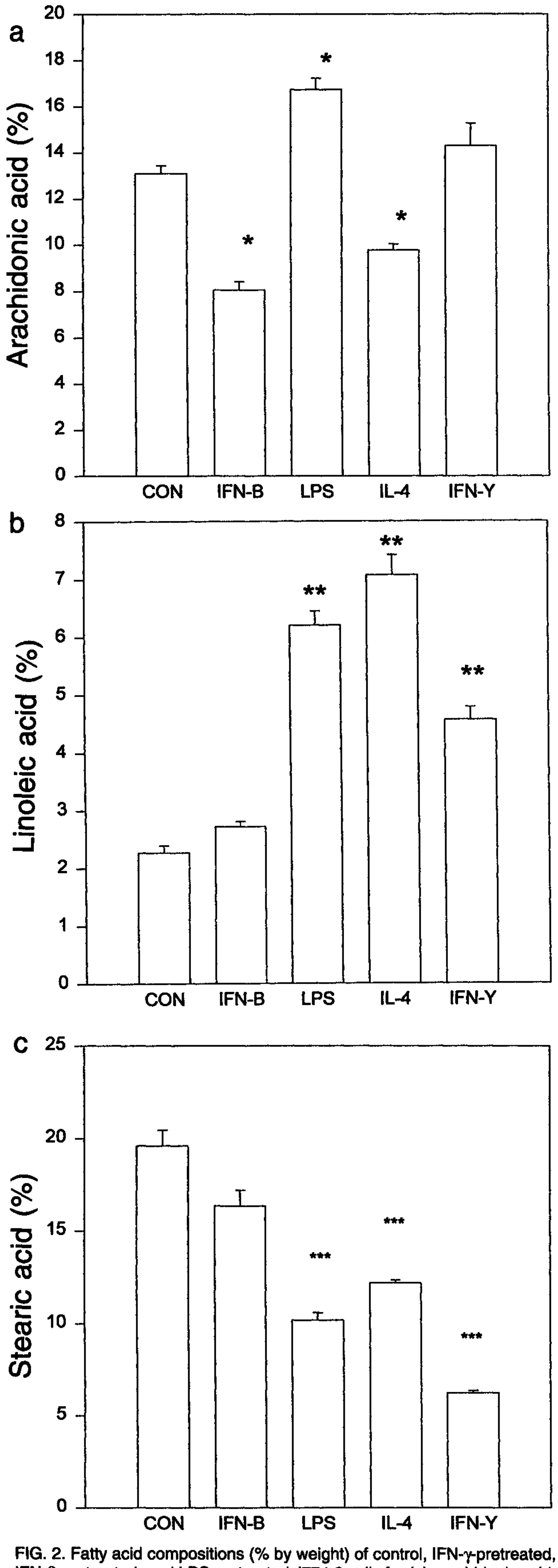

FIG. 2. Fatty acid compositions (% by weight) of control, IFN-γ-pretreated, IFN-β-pretreated, and LPS pretreated J774.2 cells for (a) arachidonic acid; (b) linoleic acid and (c) stearic acid. Cells ($1 \times 10^6$/ml) were pretreated with either 50 U/ml IFN-γ or IFN-β, or with 10 U/ml LPS for 18 h at 37°C. Error bars represent ± standard error of the means. (*$p < 0.05$; **$p < 0.01$; ***$p < 0.001$ *vs.* control cells).

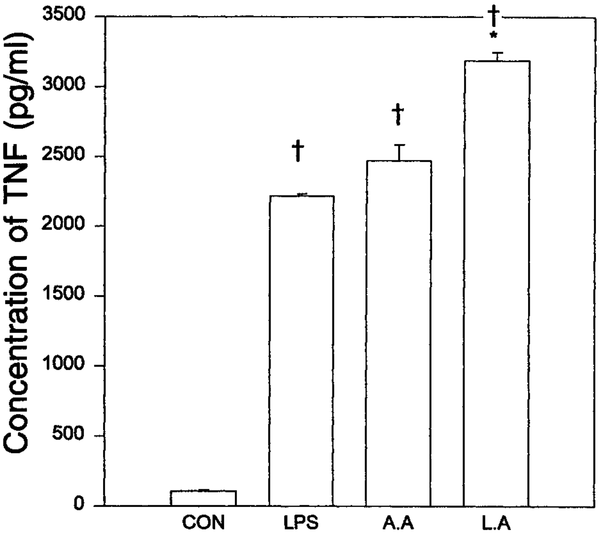

FIG. 3 TNFα release in control cells, and in control cells and cells preincubated with arachidonic (AA) and linoleic acids (LA) (50 μg/ml) for 18 h at 37°C which were subsequently incubated with LPS (100 ng/ml) for 4 h at 37°C. Error bars represent ± standard error of the means. (*$p < 0.05$ *vs.* LPS-treated cells; †$p < 0.00001$ *vs.* control cells).

quent incubation with LPS did not show increased production of TNFα.

Figure 4 shows the release of nitric oxide (measured as nitrite) from control cells and from cells pretreated with IFN-γ, IFN-β and IL-4, linoleic and arachidonic acids with or without LPS. IFN-γ and LPS on their own induced increases in the production of nitric oxide ($p < 0.00001$ *vs.* control cells for both) but this increase was much more marked in cells preincubated with IFN-γ in the presence of LPS. IFNβ did not have any effect on the production of nitric oxide. IL-4, on the other hand, induced a statistically significant inhibition in the LPS-mediated production of nitric oxide ($p < 0.05$ *vs.* LPS treated cells).

When cells were incubated with arachidonic acid and LPS together there was a slight but statistically significant decrease in nitric oxide production ($p < 0.05$ *vs.* LPS treated cells). Linoleic acid with LPS induced a statistically significant increase in nitric oxide production ($p < 0.01$ *vs.* LPS treated cells). When the cells were preincubated with linoleic acid or arachidonic acid alone there was no change in nitric oxide levels (data not shown).

### Discussion

It has previously been reported that IFN-γ may exert at least some of its effects on macrophage cells by increasing the polyunsaturation of the fatty acyl side chains of membrane phospholipids.[7] These may be important in the subsequent interaction of macrophages with endotoxin.[8] This study has shown that LPS increases the content of the polyunsaturated fatty acids, arachidonate and linoleate and decreases the content of the saturated fatty acid, stearate, in the membrane PE fraction. Interestingly, LPS-pretreated cells showed greater increases in the content of unsaturated fatty acids and greater decreases in the content of the saturated fatty acid, in comparison with IFN-γ pretreated cells.

In this study it was found that IL-4 acts similarly to IFN-γ and LPS in increasing the linoleic acid and decreasing the stearic acid content of J774.2 cells. IFN-β caused a decrease in the content of arachidonic acid (Fig. 2a) and a slight increase in the linoleic acid (Fig. 2b) content of PE in J774.2 cells. The effects of IFN-β which were found for J774.2 cells are in marked contrast to those for IFN-γ.

It has recently been reported that IFN-β induces a significant decrease in membrane lipid bilayer fluidity of J774.2 cells and the decreased fluidity is in keeping with the slightly decreased incorporation of $^{14}$C-linoleic acid in these macrophage membrane phospholipids.[18] IFN-β has also been reported to increase the saturated fatty acid content of mouse sarcoma S-180 cells.[19] These are in agreement with the effects of IFN-β that were found for murine macrophages.

It has been found that IL-4 acted similarly with LPS and IFN-γ in increasing the incorporation of $^{14}$C-linoleic acid into the PE fraction of J774.2 cell membranes. However, IFN-γ induced increases in incorporation of this fatty acid into all fractions of membrane phospholipids, whereas IL-4 induced decreases in the PC and PI fractions. The effects of IL-10 were not examined since this cytokine did not stimulate uptake of $^{14}$C-linoleic acid.

The fact that IFN-γ and LPS both increase the content of linoleic and arachidonic acids and IFN-β does not, and that IFN-γ priming can lead to enhanced LPS binding which can be mimicked by linoleic and arachidonic acids but not by interferon-β[8] also supports our hypothesis that a possible mechanism for IFN-γ-induced LPS hypersensitivity involves fatty acid changes in the membrane phospholipids.

The consequence of these lipid changes are that IFN-γ increases membrane fluidity and in so doing can render greater molecular mobility of membrane receptors. This results in increased LPS binding to IFN-γ-pretreated macrophages and also greater expression of CD-14 (unpublished results) — recently reported to be a receptor for complexes of LPS and LPS-binding protein.

Increasing evidence suggests that macrophage activation and cytokine production can be regulated by polyunsaturated fatty acids (PUFA). Thus, n-3 PUFA have been shown to suppress the ability of macrophages to produce IL-1, IL-6 and TNFα, but the n-6 PUFA, typified by linoleic acid, can enhance their production.[20] The oxidative metabolites of PUFA

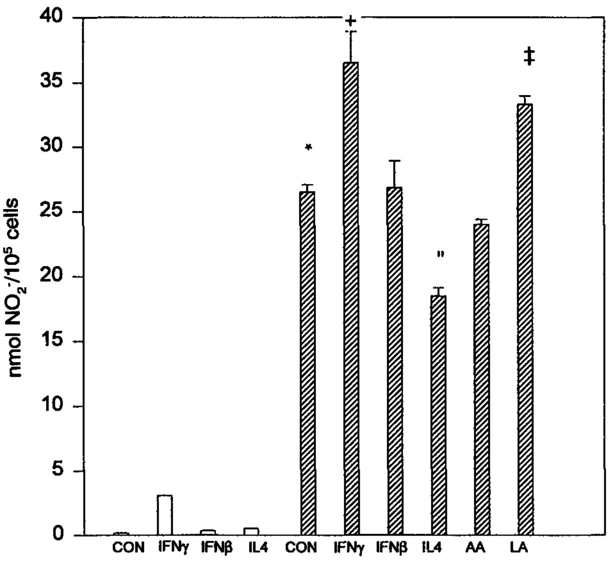

FIG. 4. Production of nitric oxide (measured as nitrite) from control cells and from cells pretreated with IFN-γ, IFN-β and IL-4, arachidonic (AA) and linoleic acids (LA) with and without 100 ng/ml LPS. Cells (1 × $10^6$/ml) were preincubated with the cytokines at a concentration of 50 U/ml, and with 50 µg/ml of arachidonic or linoleic acids for 18 h at 37°C. Error bars represent ± standard error of the means. (*$p < 0.00001$ *vs.* control cells; † $p < 0.05$ and ‡$p < 0.01$ both *vs.* LPS-treated cells). □, without LPS; ■, with LPS.

(eicosanoids) have been shown to regulate macrophage inflammatory reactions including cytokine synthesis. Among the arachidonic acid metabolites, prostaglandin $E_2$ ($PGE_2$) has been found to have important feedback actions on macrophages. In many cases $PGE_2$ provides a negative feedback signal for the production of cytokines such as TNFα[21] and IL-1.[22] Interestingly, it has been suggested that IFN-γ can down-regulate $PGE_2$ production, perhaps via a decrease in cyclooxygenase activity, and this enhances the production of the pro-inflammatory cytokines.[23]

Several recent studies provide evidence that lipoxygenases are involved in the activation of mononuclear phagocytes.[24] It is well recognized that TNF mediates many of the lethal effects of endotoxin and recently 13-hydroxylinoleic acid has been reported to be of functional importance in TNF formation by macrophages treated with LPS.[25] Pretreatment of macrophages with IFN-γ and IL-4 results in LPS-induced TNFα release and it is believed that this could be as a result of increases in the linoleic acid content of these macrophages.

From our results it can be seen that IL-4 induces increases in the linoleic acid but not the arachidonic acid content in J774.2 macrophage membranes. IL-4 decreases LPS-induced nitric oxide production but increases LPS-induced TNFα production, in agreement with recent findings.[26] Therefore, induction of TNFα in this case is not due to increases in endogenous arachidonic acid and suggests that linoleic acid may play a significant role in TNFα production by macrophages. Induction of nitric oxide release, on the other hand, may require the presence of both endogenous arachidonic and linoleic acids.

Linoleic acid, therefore, may be an important mediator in LPS-induced responses in macrophages, and IFN-γ and IL-4, by increasing the linoleate content in phospholipids, could prime these cells for a heightened response to LPS.

### References

1. Suter E, Kirsanow EM. Hyperreactivity to endotoxin in mice infected with mycobacteria. Induction of and elicitation of the reactions. *Immunol* 1961; **4**: 354–365.
2. Beutler B, Milsark IW, Cerami A. Passive immunisation against cachectin/tumor necrosis factor protects mice from lethal effect of endotoxin. *Science* 1985; **229**: 869–871.
3. Billiau A, Heremans H, Vandekerckhove F, Dillen C. Anti-interferon-γ antibody protects mice against the generalised Shwartzman reaction. *Eur J Immunol* 1987; **17**: 1851–1854.
4. Akagawa K, Tokunaga. Lack of binding of bacterial lipopolysaccharide to mouse lung macrophages and restoration of binding by interferon-γ. *J Exp Med* 1985; **162**: 1444–1459.
5. Katschinski T, Galanos C, Coumbos A, Freudenberg MA. Gamma interferon mediates *Propionibacterium acnes*-induced hypersensitivity to lipopolysaccharide in mice. *Infect Immun* 1992; **60**: 1994–2001.
6. Jackson SK, Stark JM, Taylor S, Harwood JL. Changes in phospholipid fatty acid composition and triacylglycerol content in mouse tissues after infection with bacille Calmette–Guerin. *Br J Exp Path* 1989; **70**: 435.
7. Jackson SK, Darmani H, Stark JM, Harwood JL. Interferon-γ increase macrophage phospholipid polyunsaturation: a mechanism of endotoxin sensitivity. *Int J Exp Path* 1992; **73**: 783–791.
8. Darmani H, Harwood JL, Jackson SK. Interferon-γ and PUFA increase the binding of LPS to macrophages. *Int J Exp Path* **75**: 363-368.
9. Darmani H, Jackson SK, Harwood JL. Stimulation of phospholipid unsaturated acyl turnover by interferon-γ. *Cell Immunol* 1993; **152**: 59–71.
10. Cunha FQ, Moncada S, Liew Y. Interleukin-10 (IL-10) inhibits the induction of nitric oxide synthase by interferon-gamma in murine macrophages. *Biochem and Biophys Res Commun* 1992; **182**: 1155–1159.
11. Mosmann TR, Coffman RL. Heterogeneity of cytokine secretion patterns and functions of helper T cells. *Adv Immunol* 1989; **46**: 111–147.
12. Scott P, Natovitz P, Coffman RL, Pearce E, Sher A. Immunoregulation of cutaneous leishmaniasis. T cell lines that transfer protective immunity or exacerbation belong to different T helper subsets and respond to distinct parasite antigens. *J Exp Med* 1988; **168**: 1675–1684.
13. Heinzel FP, Sadick MD, Holaday BJ, Coffman RL, Locksley RM. Reciprocal expression of interferon gamma or interleukin 4 during the resolution or progression of murine leishmaniasis. Evidence for expansion of distinct helper T cell subsets. *J Exp Med* 1989; **169**: 59–72.
14. Gajewski TF, Fitch FW. Differential activation of murine TH1 and TH2 clones. *Res Immunol* 1991; **142**: 19–23.
15. Fiorentino DF, Bond MW, Mosmann TR. Two types of mouse T helper cell. IV. Th2 clones secrete a factor that inhibits cytokine production by Th1 clones. *J Exp Med* 1989; **170**: 2081–2095.
16. Garbus J, De Luca HF, Loomans ME, Strong FM. The rapid incorporation of phosphate into mitochondrial lipids. *J Biol Chem* 1963; **238**: 59–63.
17. Stark JM, Jackson SK. Sensitivity to endotoxin is induced by increased membrane fatty acid unsaturation and oxidant stress. *J Med Microbiol* 1990; **32**: 217–221.
18. Darmani H, Harwood JL, Jackson SK. Differential effects of interferon-γ, and -β on fatty acid turnover, lipid bilayer fluidity and TNF-α release in murine macrophage J774.2 cells. *Int J Exp Path* (submitted).
19. Chandrabose K, Cuatrecasas P. Changes in fatty acyl chains of phospholipids induced by interferon in mouse sarcoma S-180 cells. *Biochem Biophys Res Commun* 1981; **98**: 661–668.
20. Grimble RF. The modulation of immune function by dietary fat. *Br J Int Care* (in press).
21. Kunkel SL, Spengler ML. Prostaglandin E2 regulates macrophage-derived tumour necrosis factor gene expression. *J Biol Chem* 1988; **263**: 5380–5384.
22. Kunkel SL, Chensue SW. Arachidonic acid metabolites regulate interleukin-1 production. *Biochem Biophys Res Commun* 1985; **128**: 892–897.
23. Boraschi D, Censini S, Tagliabue. Interferon-γ reduces macrophage-suppressive activity by inhibiting prostaglandin E2 release and inducing interleukin-1 production. *J Immunol* 1984; **133**: 764–768.
24. Schade UF. Involvement of lipoxygenases in the activation of mouse peritoneal macrophages by endotoxin. *Biochem Biophys Res Commun* 1986; **138**: 842–849.
25. Schade UF, Engel R, Jacobs D. Differential protective activities of site specific lipoxygenase inhibitors in endotoxic shock and production of tumor necrosis factor. *Int J Immunopharm* 1991; **13**: 565–571.
26. Suk K, Somers SD, Erickson KL, Regulation of murine macrophage function by IL-4: IL-4 and IFN-gamma differentially regulate macrophage tumoricidal activation. *Immunol* 1993; **80**: 617–624.

ACKNOWLEDGEMENT. The financial support of the Medical Research Council is gratefully acknowledged.

**Received 2 August 1994; accepted in revised form 22 September 1994**
